# Fibroblast Growth Factor 19 Levels Predict Subclinical Atherosclerosis in Men With Type 2 Diabetes

**DOI:** 10.3389/fendo.2020.00282

**Published:** 2020-05-22

**Authors:** Jingyi Hu, Zhiwen Liu, Yue Tong, Zubing Mei, Aimin Xu, Pengcheng Zhou, Xiaoyan Chen, Weili Tang, Zhiguang Zhou, Yang Xiao

**Affiliations:** ^1^National Clinical Research Center for Metabolic Diseases, Department of Metabolism and Endocrinology, The Second Xiangya Hospital, Central South University, Changsha, China; ^2^Department of Endocrinology, Xuhui District Central Hospital, Shanghai, China; ^3^Department of Anorectal Surgery, Shuguang Hospital, Shanghai University of Traditional Chinese Medicine, Shanghai, China; ^4^Anorectal Disease Institute of Shuguang Hospital, Shanghai, China; ^5^State Key Laboratory of Pharmaceutical Biotechnology, The University of Hong Kong, Hong Kong, China; ^6^Department of Medicine, The University of Hong Kong, Hong Kong, China; ^7^Department of Pharmacology and Pharmacy, The University of Hong Kong, Hong Kong, China; ^8^Research Center of Heart, Brain, Hormone, and Healthy Aging, The University of Hong Kong, Hong Kong, China

**Keywords:** fibroblast growth factor 19, atherosclerosis, intima-media thickness, type 2 diabetes, prediction

## Abstract

**Objective:** Fibroblast growth factor 19 (FGF19) plays an indispensable role in regulating bile acid, glucose, and lipid metabolism, and alterations of its circulating concentration is associated with the development of type 2 diabetes (T2D). Atherosclerosis is directly related to the death-deriving diabetic macroangiopathy in T2D, yet relationships between FGF19 and atherosclerosis in T2D remain unclear. The aim of this study was to investigate the association of circulating FGF19 levels with the development of subclinical atherosclerosis (subAS) in patients with T2D in a 3-year prospective study.

**Methods:** In the present study, 153 newly diagnosed T2D patients without subAS were recruited at baseline, and 137 of them completed a 3-year follow-up. FGF19 levels were measured in fasting serum samples collected at baseline and the third-year visits. Carotid, femoral, and iliac intima-media thickness (IMT) were detected by high-resolution B-mode ultrasound to determine the presence of subAS. Logistic regression analysis was applied to assess the relationship between serum FGF19 and subAS in patients with T2D.

**Results:** At baseline, serum FGF19 levels were positively correlated with carotid IMT and iliac IMT in men (*r* = 0.239, *P* = 0.036; *r* = 0.309, *P* = 0.006). At the 3-year follow-up, 25 out of 153 patients developed subAS, and FGF19 levels in men were higher in the subAS group than in the non-subAS group [202.7 (177.9–373.6) vs. 133.4 (85.6–171.3) pg/ml, *P* = 0.028]. Furthermore, in men, higher baseline levels of FGF19 were independently associated with a greater risk of subAS at year 3 in patients with T2D with an odds ratio (OR) of 4.798 per 1 standard deviation (SD) of the FGF19 concentration [OR = 4.798 (95% CI, 1.680–13.706), *P* = 0.003]. Baseline FGF19 levels yielded an area under the receiver operating characteristic curve of 0.769 to predict the development of subAS at year 3 in men with T2D.

**Conclusions:** Serum FGF19 levels could help in predicting the development of atherosclerosis in men with T2D.

## Introduction

Diabetes mellitus is currently burdening approximately 463 million people worldwide with booming healthcare cost and plummeting disability adjusted life year (1–3). Among them, nearly 90% were with type 2 diabetes (T2D) (1). Diabetic macroangiopathy, which is characterized by atherosclerosis, is the most severe diabetic complication, which makes major contributions to diabetic mortality and morbidity (4). Atherosclerosis is the reformation of blood vessels through the accumulation of lipids and redox signaling induction of factors in the arterial wall, which could result in rupture and stenosis in arteries and subsequent death-deriving processes (5). Subclinical atherosclerosis (subAS) is an early indicator of the development of atherosclerosis, which could be assessed by increased intima-media thickness (IMT) of arteries. This early identification could be conducive to early intervention and treatment, thus preventing further development of cardiovascular disease (6, 7).

Fibroblast growth factor 19 (FGF19) is a circulating hormone that actively participates in governing bile acid synthesis, glycogenesis, and lipid metabolism (8, 9). Given these functions, FGF19 is a potential molecular in investigating human chronic disease, including T2D and atherosclerosis, in which glucose and lipid metabolism play a vital role (9, 10). In this regard, a recent study conducted on C57BL/6 and FGF19-knockout mice found that the expression of FGF19 could inhibit the absorption of cholesterol in the liver (11).

In human, scientists have revealed a negative correlation between FGF19 and cardiovascular risk factors (12) and found FGF19 as an independent factor of the development of coronary artery disease (13). The concentration of FGF19 was negatively correlated with the concentration of triglycerides (TG) and the plasma atherosclerosis index but was positively correlated to high-density lipoprotein cholesterol (HDL-c), which proved the vital importance of FGF19 in lipid metabolism and atherosclerosis (12–14). Based on these results, FGF19 analogs were applied in clinical trials to evaluate its potential in modulating lipid metabolism, and results revealed increased serum cholesterol, high-density lipoprotein cholesterol (HDL-C), low-density lipoprotein cholesterol (LDL-C), and reduced TG levels in human participants (15). However, the relationship between FGF19 and diabetic macroangiopathy has not been demonstrated extensively. In this study, we investigated serum concentration of FGF19 and early atherosclerosis events in human T2D patients to assess whether serum FGF19 could be applied in predicting atherosclerosis in T2D.

## Methods

### Participants

Participants were recruited in the Chinese National Tenth and Eleventh Five Tackling Key Project from 2002 to 2007 at the Second Xiangya Hospital of Central South University. In this prospective study, 153 newly diagnosed T2D patients were defined by the World Health Organization (WHO) and American Diabetes Association (ADA) criteria (16, 17). Patients with (1) other types of diabetes (as defined by the ADA classification), (2) overt cardiovascular and cerebrovascular diseases (including angina, myocardial infarction, stroke, peripheral vascular disease), (3) severe liver and kidney dysfunction, (4) tumors, and (5) severe trauma or surgery were excluded. The diagnosis of dyslipidemia was based on the 2001 American National Cholesterol Education Program Adult Treatment Panel III (NECP-ATPIII) standard (18). Hypertension was diagnosed when systolic blood pressure (SBP) ≥140 or diastolic blood pressure (DBP) ≥90 mmHg, or if patients were taking antihypertensive medications. Smoking referred to both current and past smokers. Alcohol consumption referred to both current and past alcohol consumption. Metformin was prescribed for patients with a body mass index (BMI) of 25 kg/m^2^ or above, and glipizide was recommended for patients with a BMI <25 kg/m^2^ (19). This study was carried out in accordance with the Helsinki Declaration of the World Medical Association and approved by the Ethics Committee of the Second Xiangya Hospital of Central South University. Written informed consent was obtained from each subject upon recruitment.

### Clinical and Biochemical Assessments

Age, sex, and medical history of each patient were recorded, and their height, weight, and waist and hip circumference were measured with a standard procedure. Subjects were asked to undertake 8 h fasting before the visit in early morning, and venous blood was collected. Separated serum was used to detect biochemical parameters, and the remaining specimens were stored at −80°C for FGF19 detection. Plasma glucose, serum cholesterol, TG, HDL-C, LDL-C, and high-sensitivity C-reactive protein (hsCRP) were measured on a Hitachi 7170 analyzer (Boehringer Mannheim, Mannheim, Germany). Serum insulin was measured by Bayer 180SE's automated chemiluminescence system (Bayer AG, Leverkusen, Germany) (20). Insulin resistance (IR) was assessed using the homeostasis model assessment of insulin resistance (HOMA-IR). The plasma atherosclerosis index (AIP) was calculated as log (triglyceride/high-density lipoprotein cholesterol) (13). Serum FGF19 were measured with an enzyme-linked immunosorbent assay (ELISA) kit (The University of Hong Kong Antibody and Immunoassay Service), and the intra- and interassay precision met the manufacturer's instructions (19).

### Intima-Media Thickness Assessment

Doppler vascular ultrasound examination was performed annually on all patients with the Doppler ultrasound system (128XP/10 system; Acuson, Mountain View, CA, USA) (21). The IMT of the patient's carotid, femoral, and iliac arteries were measured, and intra- and interassay coefficients of variance were 3.9–4.2% and 5.0–6.0%, respectively. Patients with one or more arteries with an IMT >1.0 mm or plaque formation without clinical manifestations were considered with subAS (22, 23).

### Statistical Analysis

Statistical analyses were performed with SPSS 25.0 software and GraphPad Prism 8.0 software. Normally distributed data determined by Kolmogorov–Smirnov test were presented as mean ± standard deviation (SD). Non-normally distributed data were presented as median and interquartile range (IQR). Comparisons between groups were performed using χ^2^ tests for categorical variables. Unpaired Student's *t*-tests and non-parametric tests were used to compare differences in normally distributed and non-normally distributed data between two groups. Spearman correlation analysis was performed to analyze the correlation between serum FGF19 levels and other variables. The values of serum FGF19 were standardized, and logistic regression analysis was applied to investigate the association between the change in serum FGF19 level per 1 standard deviation (SD) and subAS in men with T2D. The area under the curve (AUC) of baseline FGF19 levels or FGF19 levels at year 3 was calculated to evaluate the predictive value or the diagnostic performance by receiver operating characteristic curve (ROC) analysis. Two-sided *P* < 0.05 was considered statistically significant.

## Results

Baseline characteristics of a total of 153 patients with T2D (77 men and 76 women) are summarized in Table 1. No significant differences were shown in age, BMI, fasting blood glucose (FBG), 2-h plasma glucose (2hPG), LDL-c, total cholesterol (TC), blood urea nitrogen (BUN), fasting insulin (FINS), SBP, DBP, HOMA-IR, femoral IMT, and iliac IMT between men and women (*P* > 0.05). Hemoglobin A1c (HbA1c) of all participants was 7.6 (6.0–9.4)%, suggesting overall hyperglycemia. Compared with women, men were with higher waist/hip ratio (WHR), HbA1c, alanine transaminase (ALT), total bilirubin (TBIL), TG, 24-h urine microalbumin (24hUALB), AIP, percentage of smoking, percentage of alcohol consumption, and carotid IMT, but lower HDL-c levels. No statistically significant differences in baseline serum FGF19 levels between men and women were observed [131.4 (76.4–193.5) vs. 150.4 (90.6–280.2) pg/ml, *P* = 0.61]. However, men had greater carotid IMT than women.

**Table 1:** Anthropometric parameters and clinical characteristics among subjects.

	**All patients (*n* = 153)**	**Women (*n* = 76)**	**Men (*n* = 77)**	***P*-values**
Age (years)	54.0 (48.5–61.0)	55.0 (52.0–60.8)	52.0 (46.0–61.0)	NS
BMI (kg/m^2^)	23.9 (22.7–25.8)	23.7 (22.6–25.4)	24.3 (23.1–26.0)	NS
WHR	0.89 ± 0.06	0.86 ± 0.05	0.92 ± 0.05	<0.001
FBG (mmol/L)	7.0 (5.9–8.0)	7.1(6.1–8.0)	6.9 (5.7–8.2)	NS
2hPG (mmol/L)	11.3 (8.3–14.7)	11.2 (8.4–14.3)	11.3 (7.9–15.3)	NS
HbA1c (%)	7.6 (5.9–9.4)	6.8 (5.9–8.5)	8.1 (6.4–9.7)	0.018
HDL-c (mmol/L)	1.3 ± 0.3	1.4 ± 0.3	1.2 ± 0.3	<0.001
LDL-c (mmol/L)	3.0 ± 0.9	3.0 ± 0.8	3.0 ± 1.0	NS
ALT (U/L)	24.8 (17.9–36.4)	21.8 (15.7–32.3)	26.0 (20.9–38.9)	0.024
TBIL (μmol/L)	13.3 (11.0–18.2)	12.1 (9.9–15.9)	15.4 (11.7–19.0)	0.028
BUN (mmol/L)	5.3 (4.4–6.4)	5.4 (4.4–6.5)	5.3 (4.4–6.4)	NS
Cr (μmol/L)	94.4 ± 17.5	87.7 ± 12.5	101.0 ± 19.0	<0.001
TG (mmol/L)	1.7 (1.2–2.7)	1.6 (1.1–2.2)	2.0 (1.3–3.3)	0.022
TC (mmol/L)	5.2 ± 1.1	5.2 ± 0.9	5.2 ± 1.2	NS
24hUALB (mg/24 h)	55.7 (32.7–104.1)	47.2 (25.9–87.8)	70.4 (41.0–116.5)	0.017
FINS (μU/ml)	14.0 (9.0–19.0)	13.0 (9.0–21.0)	14.0 (10.0–18.0)	NS
SBP (mmHg)	120.0 (110.0–130.0)	120.0 (105.0–135.0)	120.0 (110.0–129.0)	NS
DBP (mmHg)	75.0 (75.0–84.8)	75.0 (70.0–80.0)	80.0 (70.0–85.0)	NS
HOMA-IR	4.6 (2.7–6.5)	4.5 (2.7–6.9)	4.6 (2.8–6.4)	NS
AIP	2.1 (1.9–2.4)	2.1 (1.8–2.2)	2.2 (2.0–2.5)	0.001
Smoking, *n* (%)	62 (40.5%)	2 (2.6%)	60 (77.9%)	<0.001
Alcohol consumption, *n* (%)	44 (28.8%)	4 (5.3%)	40 (51.9%)	<0.001
Carotid IMT (mm)	0.70 (0.60–0.80)	0.67 (0.60–0.77)	0.73 (0.64–0.80)	0.003
Femoral IMT(mm)	0.73 (0.60–0.80)	0.73 (0.60–0.80)	0.73 (0.63–0.80)	NS
Iliac IMT (mm)	0.79 (0.71–0.80)	0.80 (0.70–0.80)	0.77 (0.72–0.80)	NS
FGF19^*^ (pg/ml)	133.5 (81.6–219.2)	147.8 (90.6–283.0)	131.4 (76.4–193.5)	NS

* *Log transformed before analysis. Data are means ± SD or IQR*.

*BMI, body mass index; WHR, waist/hip ratio; FBG, fasting blood glucose; 2hPG, 2-h post-prandial blood glucose; HbA1c, hemoglobin A1c; HDL-c, high-density lipoprotein cholesterol; LDL-c, low-density lipoprotein cholesterol; ALT, alanine transaminase; TBIL, total bilirubin; BUN, blood urea nitrogen; Cr, creatinine; TC, total cholesterol; TG, triglycerides; 24hUALB, 24-h urine microalbumin; FINS, fasting insulin; SBP, systolic blood pressure; DBP, diastolic blood pressure; HOMA-IR, homeostasis model assessment of insulin resistance; AIP, plasma atherosclerosis index; carotid IMT, carotid intima-media thickness; femoral IMT, femoral intima-media thickness; iliac IMT, iliac intima-media thickness; FGF19, fibroblast growth factor 19; SD, standard deviation; IQR, interquartile range*.

**Figure 1:**
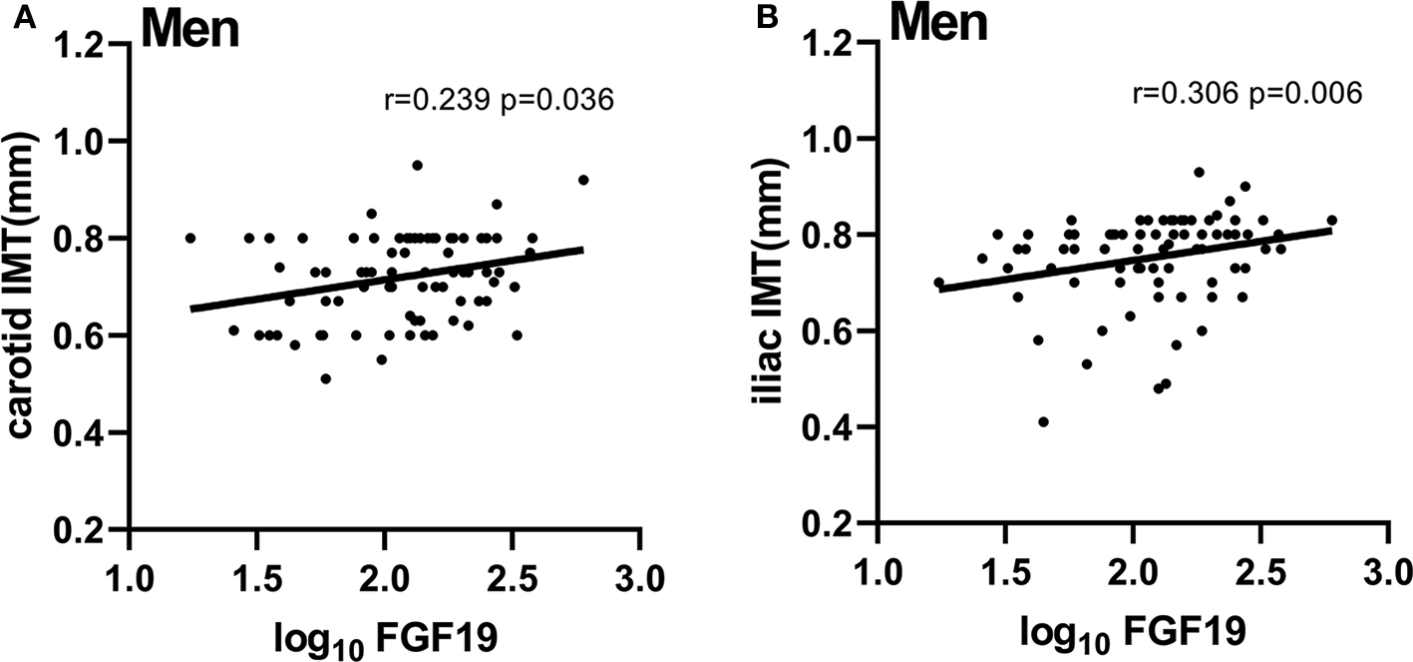
Serum FGF19 levels were positively correlated with carotid IMT and iliac IMT in men. **(A)** Correlation of FGF19 and carotid IMT in men, carotid IMT = 0.07958 × log_10_FGF19 + 0.5549. **(B)** Correlation of FGF19 and iliac IMT in men, iliac IMT = 0.07939 × log_10_FGF19 + 0.5873. FGF19, fibroblast growth factor 19; carotid IMT, carotid intima-media thickness; iliac IMT, iliac intima-media thickness; log_10_ FGF19, log10 transformed FGF19 levels.

Correlations between baseline serum FGF19 levels and baseline characteristics are shown in Table 2. Among all participants, serum levels of FGF19 were negatively correlated with body weight, FINS, and HOMA-IR (*r* = −0.181, *P* = 0.026; *r* = −0.189, *P* = 0.020; *r* = −0.163, *P* = 0.045) but positively correlated with iliac IMT (*r* = 0.208, *P* = 0.010). We then assessed these associations based on a sex-specific manner. In women, we only found the FGF19 level to be positively correlated with WHR (*r* = 0.248, *P* = 0.032). In men, FGF19 levels were negatively correlated with BMI, FINS, and HOMA-IR (*r* = −0.251, *P* = 0.022; *r* = −0.388, *P* = 0.001, *r* = −0.353, *P* = 0.002). In addition, we found significant correlations between serum FGF19 levels and carotid IMT, iliac IMT in men (*r* = 0.239, *P* = 0.036; *r* = 0.309, *P* = 0.006; Figure 1). Partial correlation analysis adjusted for age and BMI showed that positive correlations between FGF19 and IMT were still statistically significant (*r* = 0.313, *P* = 0.006; *r* = 0.285, *P* = 0.013).

**Table 2:** Spearman correlations between serum FGF19 levels and various physiological and clinical indicators.

	**All patients**		**Women**		**Men**	
	***r***	***P*-value**	***r***	***P*-value**	***r***	***P*-value**
Age (years)	0.063	0.439	−0.091	0.436	0.178	0.122
BMI (kg/m^2^)	−0.095	0.243	0.093	0.428	−0.251*	0.022
Body weight (kg)	−0.181*	0.026	−0.201	0.080	−0.037	0.752
WHR	0.037	0.650	0.248*	0.032	0.052	0.652
FBG (mmol/L)	−0.044	0.594	0.089	0.447	−0.186	0.106
2hPG (mmol/L)	−0.092	0.304	−0.020	0.875	−0.190	0.136
HbA1c (%)	−0.102	0.211	−0.085	0.468	−0.063	0.587
HDL-c (mmol/L)	0.093	0.257	0.013	0.912	0.065	0.558
LDL-c (mmol/L)	−0.020	0.803	0.002	0.985	−0.109	0.325
ALT (U/L)	0.005	0.956	0.184	0.113	−0.140	0.224
TBIL (μmol/L)	−0.148	0.070	−0.101	0.395	−0.133	0.247
BUN (mmol/L)	0.089	0.279	0.006	0.960	0.183	0.112
Cr (μmol/L)	−0.133	0.105	−0.228	0.050	0.043	0.710
TG (mmol/L)	−0.087	0.287	0.086	0.461	−0.206	0.072
TC (mmol/L)	−0.027	0.742	0.091	0.437	−0.131	0.258
24hUALB (mg/24 h)	0.008	0.924	0.040	0.734	0.004	0.975
FINS (μU/mL)	−0.189*	0.020	−0.004	0.970	−0.388**	0.001
HOMA-IR	−0.163*	0.045	0.007	0.949	−0.353**	0.002
AIP	−0.106	0.197	0.065	0.583	−0.206	0.073
SBP (mmHg)	−0.099	0.226	−0.106	0.365	−0.113	0.328
DBP (mmHg)	−0.130	0.109	−0.055	0.640	−0.182	0.113

*BMI, body mass index; WHR, waist/hip ratio; FBG, fasting blood glucose; 2hPG, 2-h post-prandial blood glucose; HbA1c, hemoglobin A1c; HDL-c, high-density lipoprotein cholesterol; LDL-c, low-density lipoprotein cholesterol; ALT, alanine transaminase; TBIL, total bilirubin; BUN, blood urea nitrogen; Cr, creatinine; TC, total cholesterol; TG, triglycerides; 24hUALB, 24-h urine microalbumin; FINS, fasting insulin; HOMA-IR, homeostasis model assessment of insulin resistance; AIP, plasma atherosclerosis index; SBP, systolic blood pressure; DBP, diastolic blood pressure*.

Of the 153 patients, none were lost during the first year of follow-up, 4 were lost to follow-up in the second year, and 12 were lost to follow-up in the third year. The main reasons for their loss of follow-up were changes in patients' mobile numbers, changes in medication, lack of consent, removal, or death. There was no statistically significant difference in baseline data between patients who were lost during the follow-up and those who were not, except that the patients who were lost to follow-up were younger and had lower serum Cr (Supplementary Table 2). However, all participants had no serious kidney disease. We then assessed this difference based on a sex-specific manner, and the results were consistent with those of all participants. Twenty-five of the 153 (16.3%) participants developed subAS at the end of the 3-year follow-up (Supplementary Table 1).

In men, in order to investigate the association between serum FGF19 levels and the development of subAS, unadjusted and multivariable adjusted odds ratios (ORs) for per 1-SD increase in baseline serum FGF19 level are presented in Table 3. Multiple logistic regression analysis was conducted with the diagnosis of subAS at year 3 as the dependent variable and per 1-SD increase in baseline serum FGF19 level, age, BMI, HbA1c, smoking, alcohol consumption, presence of hypertension, and presence of dyslipidemia as independent variables. Per 1-SD increase in serum FGF19 level was significantly associated with a 4.798-fold increased risk of subAS in men with T2D (Table 3). Furthermore, per 1-SD increase in serum FGF19 level at year 3 was significantly associated with a 2.987-fold increased risk of subAS in men with T2D (Supplementary Table 3).

**Table 3:** Logistic regression analysis showing baseline FGF19 concentrations independently associated with the subAS at year 3 in men.

	**Simple**		**Multiple**	
	**OR (95%CI)**	***P***	**OR (95%CI)**	***P***
FGF19 (per 1-SD increase)	3.221 (1.500–6.918)	0.003	4.798 (1.680–13.706)	0.003
Age	1.049 (0.977–1.127)	0.183	1.072 (0.964–1.192)	0.200
BMI	0.768 (0.584–1.010)	0.059	0.814 (0.587–1.129)	0.218
Smoking	1.344 (0.260–6.962)	0.724	7.104 (0.432–113.944)	0.171
Alcohol consumption	1.167 (0.348–3.911)	0.803	0.250 (0.028–2.258)	0.217
HbA1c	1.071 (0.814–1.409)	0.626	1.123 (0.735–1.715)	0.592
Presence of hypertension	2.292 (0.633–8.299)	0.207	9.118 (1.212–68.611)	0.032
Presence of dyslipidemia	1.196 (0.229–6.254)	0.832	0.797 (0.112–5.658)	0.218

*Variables included in the multiple logistic regression model are FGF19 (per 1-SD increase), age, BMI, HbA1c, smoking, alcohol consumption, presence of hypertension, and dyslipidemia*.

*BMI, body mass index; HbA1c, hemoglobin A1c; SBP, systolic blood pressure; DBP, diastolic blood pressure; FGF19, fibroblast growth factor 19; SD, standard deviation; subAS, subclinical atherosclerosis*.

**Figure 2:**
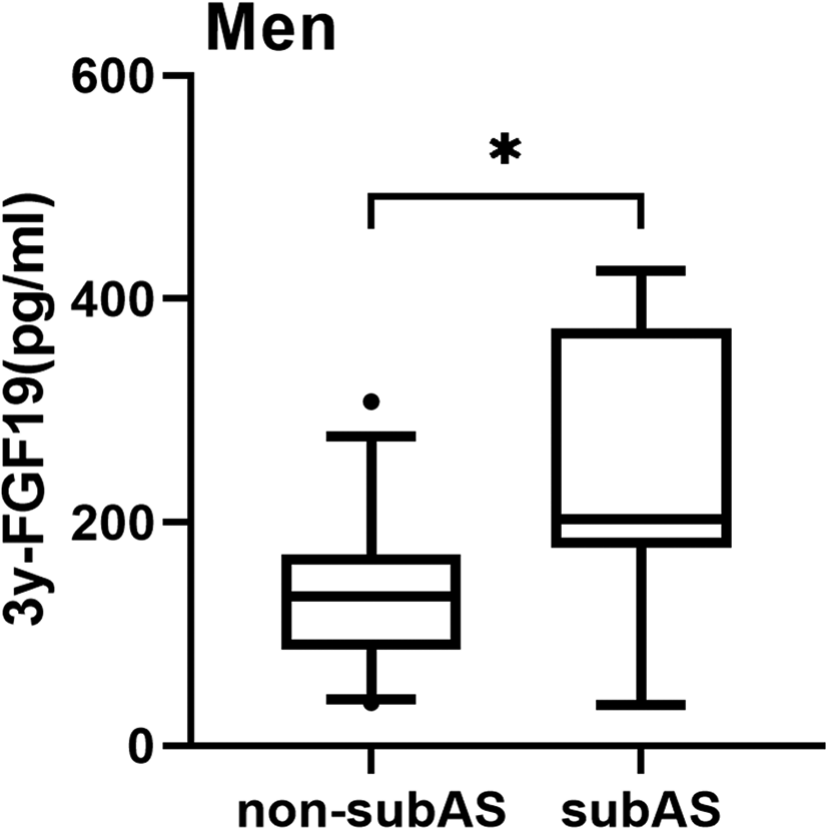
Box-and-whisker plot showed a comparison of serum levels of FGF19 at year 3 in the non-subAS group and subAS group in men. In this figure, the horizontal lines inside the boxes represent the median; the upper and lower horizontal boundaries of the boxes represent the 25th and 75th percentiles, respectively, and the upper and lower horizontal lines outside the boxes represent the 95th and 5th percentiles, respectively. *P < 0.05.

By the third year, serum FGF19 levels of all participants were 171.2 (101.6–250.4) pg/ml. Among all participants, there was no statistical difference in FGF19 levels between subjects with subAS and subjects without subAS [187.6 (120.8–309.5) vs. 166.3 (98.9–244.9) pg/ml, *P* > 0.05]. In men, FGF19 levels at year 3 were higher in the subAS group than in the non-subAS group [202.7 (177.9–373.6) vs. 133.4 (85.6–171.3) pg/ml, *P* = 0.028; Figure 2]. Baseline serum FGF19 levels in men who developed subAS by year 3 were higher than those who did not [215.6 (148.9–297.6) vs. 133.4 (68.9–165.6) pg/ml, *P* = 0.003]. We further divided patients who developed subAS into two groups with the cutoff value of BMI at 25 kg/m^2^. No significant difference in the development of subAS was observed between groups of patients with BMI higher or lower than 25 kg/m^2^ (*P* > 0.05).

**Figure 3:**
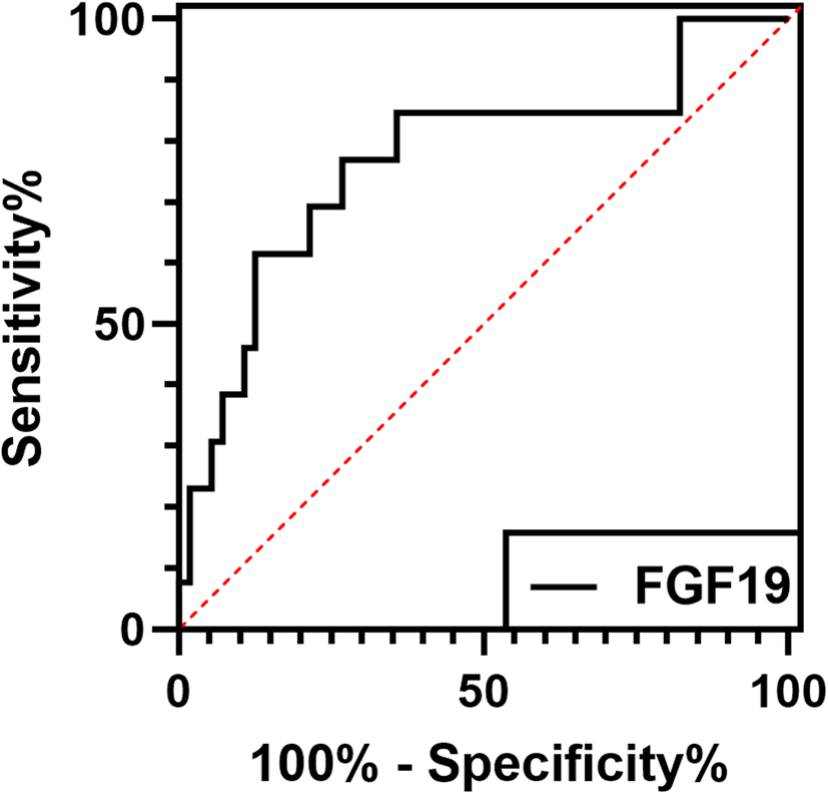
The receiver operating characteristic (ROC) curve showed that FGF19 had a high predictive value of subAS in men. The serum FGF19 levels in the baseline group had an area under the curve (AUC) of 0.769 in men.

In men, baseline FGF19 levels attained a good predictive value for the development of subAS, with an area under the receiver operating characteristic curve (AUC) of 0.769 (Youden index = 0.501, cutoff value = 156.18 pg/ml, sensitivity = 77%, specificity = 73%; Figure 3). In addition, using the FGF19 levels at year 3 yielded an AUC of 0.770 (Youden index = 0.643, cutoff value = 174.66 pg/ml, sensitivity = 86%, specificity = 79%; Supplementary Figure 1).

## Discussion

In our study, we found that serum levels of FGF19 were positively correlated with carotid IMT and iliac IMT in men. Serum FGF19 levels were higher in men who developed subAS than those who did not. High serum FGF19 levels were associated with an increased risk of subAS in men with T2D.

Patients with T2D are characterized by high blood glucose and insulin resistance as well as accompanied with obesity and dyslipidemia (24, 25). Long-term complications, especially those resulted from diabetic macroangiopathy, are intimately associated with dyslipidemia and atherosclerosis and are death-deriving events during the T2D natural history (4). These make unraveling mechanisms involved in metabolism helpful in understanding diabetic macroangiopathy and ultimately improving the prognosis of T2D. FGF19, a hormone mainly secreted by the ileum, plays a role in the bile acid cycle, regulating lipid, and glucose metabolism (26–28). Previous studies have demonstrated the critical link between these metabolic processes and human chronic diseases including T2D and atherosclerosis and suggested FGF19 as an independent factor for T2D and coronary artery disease (13, 29). Nevertheless, how FGF19 functions in atherosclerosis during the development of T2D is still unclear.

Previous studies found that patients with coronary artery disease had lower levels of FGF19 compared to healthy controls, and FGF19 may be a protective factor for severe coronary artery disease (13). Wong et al. found that FGF19 is related to the occurrence of major cardiovascular adverse events (30). Our results revealed that FGF19 was an early predictor for atherosclerosis in men. One possible explanation is the effect of FGF19 on reducing triglycerides. Dyslipidemia is a significant risk factor for atherosclerosis, and increased TG is an independent factor for atherosclerosis (14). FGF19 can reduce TG by increasing fatty acid oxidation (31). Another possible explanation is that patients with cardiovascular disease have reduced hepatic responsiveness to FGF19, leading to a compensatory increase in serum FGF19 levels, which is consistent with an increased risk of cardiovascular disease (30, 32). In addition, no correlation was detected between FGF19 and femoral IMT in this study. The reason may be that the hemodynamic environment of the arteries in different locations is different, which causes the wall shear stress, hydrostatic pressure, and periodic strains in different parts of the body to be different. Therefore, atherosclerosis formation in each artery is not consistent (33).

Although FGF19 plays an important role in bile acid synthesis in the liver, the molecular mechanism of FGF19 cross-talking with the cholesterol metabolism pathway is not fully understood (8, 26). Most of the cholesterol catabolism in the human body is converted into bile acid, which is a ligand for farnesoid X receptor (FXR) (34). FXR can induce the production of FGF19 (35). Previous studies have shown that FGF15 (FGF19 in human) can inhibit the expression of hepatic paraoxonase-1 (PON-1) in mice (36) and that human liver cancer cells treated with recombinant human FGF19 can inhibit the expression of PON-1 (37). PON-1 is an HDL-related hydrolase (38). Recent studies have found that PON-1 can prevent atherosclerosis through direct and indirect approaches (38–40). Therefore, FGF19 may promote atherosclerosis by inhibiting the protective effect of PON-1. In this study, it was found that FGF19 was an independent factor for the development of subAS, and more research is needed to clarify its specific mechanism. The range of atherosclerotic lesions in the aorta of ApoE^−/−^ mice with dyslipidemia was significantly reduced in the treatment with NGM282 (the analog of FGF19) (15). FGF19 can increase liver expression of ATP binding cassette transporter G5 (ABCG5) and ATP binding cassette transporter G8 (ABCG8), which affect cholesterol transport, to reduce the accumulation of liver cholesterol (41). In addition, FGF19 can be induced by FXR to inhibit bile acid production (35). Previous studies have reported that bile acids can promote the adhesion of monocytes and endothelium (42) and can directly affect the immune regulation of macrophages and the proliferation and migration of smooth muscle cells on the vessel wall, which is the key to the development of atherosclerotic diseases (15, 43, 44). A hepatic FGF15/19-Src-FXR phosphorylation signal cascade pathway, which was recently identified by Byun et al., has been proven to regulate cholesterol homeostasis and reduce the risk of atherosclerosis (45). We speculate that the elevated FGF19 levels in subclinical atherosclerotic patients may be secondary compensatory responses compared to non-subclinical atherosclerotic patients.

In the study, we found that serum FGF19 levels were only associated with subAS in men but not in women. It could be directly related to the sex-specific, in this condition male-specific, changes made by FGF19 on atherosclerosis. Women have smaller arterial diameters and fewer plaques (46, 47). Men have more atherosclerotic risk factors than women, such as smoking and alcohol, which is in line with our findings (46). The incidence of atherosclerosis is higher in men than in women at the same age (48), and gender differences could be attributed to differences in sex hormones between men and women (49, 50). Compared to men, women catabolize less cholesterol through bile acid synthesis (51). Estrogen can affect the synthesis of bile acids by affecting the activity of cytochrome P450 family 7 subfamily A member 1 (CYP7A1), and the concentration of chenodeoxycholic acid (CDCA) in bile acids of women is reduced, which may affect the promotion of bile acids in the intestinal tract to the expression of FGF19 (51). Further research is needed to investigate the mechanisms by which estrogen and androgen affect bile acid synthesis.

Our study has several limitations. First, the sample size of the study is relatively small. Indeed, with an alpha error of 5%, the calculated power for our study was <80% to detect the correlation between FGF19 and IMT in women, which may have limited the power to detect the significance between FGF19 and subAS in women. Thus, the predictive role of FGF19 for subAS remains to be validated by larger-scale studies, especially in women. Second, due to a limited follow-up period, the incidence of subAS is low to a certain extent. Third, although our data might suggest a causal relationship between circulating FGF19 levels and the development of subAS, further basic studies are warranted to examine the role of FGF19 in the pathogenesis of atherosclerosis. Fourth, the absence of normal control subjects without T2D in this study may prevent the results from being directly applicable to the general population.

## Conclusion

In conclusion, we have demonstrated that high circulating FGF19 levels predicted the development of subclinical atherosclerosis in men with type 2 diabetes in this Chinese diabetes cohort. The significance of these exciting data on a new biomarker for the subAS remains to be confirmed by larger long-term follow-up studies involving other ethnic populations and correlations with other cardiovascular end points.

## Data Availability Statement

The datasets generated for this study are available on request to the corresponding author.

## Ethics Statement

The studies involving human participants were reviewed and approved by Ethics Committee of the Second Xiangya Hospital of Central South University. The patients/participants provided their written informed consent to participate in this study.

## Author Contributions

JH and ZL performed the experiments and analyzed the data. JH wrote the manuscript. YT edited the manuscript. ZM helped with the statistical analysis. AX and PZ provided the ELISA kits. XC and WT collected the samples. YX and ZZ designed the study and revised the manuscript. All authors were involved in the interpretation of data, and approved the final version of the manuscript.

## Conflict of Interest

The authors declare that the research was conducted in the absence of any commercial or financial relationships that could be construed as a potential conflict of interest.
