# Supplementary material for: Fibroblast Growth Factor 19 Levels Predict Subclinical Atherosclerosis in Men With Type 2 Diabetes
Source: Front Endocrinol (Lausanne). 2020 May 22;11:282. doi: 10.3389/fendo.2020.00282 (PMC7258879; doi:10.3389/fendo.2020.00282)

**Supplementary Table 1.** Baseline clinical parameters of subjects with or without development of subAS by year 3

|  | Non-subAS (n=112) | subAS (n=25) | *P* values |
| --- | --- | --- | --- |
| Sex (male) | 56 (50.0%) | 13 (52.0%) | NS |
| Age (years) | 53.0 (49.0-59.0) | 59.0 (51.5-62.0) | NS |
| BMI (kg/m2) | 23.9 (22.7-25.9) | 23.5(22.1-24.7) | NS |
| WHR | 0.89± 0.06 | 0.90 ± 0.05 | NS |
| FPG (mmol/L) | 6.9(5.9-7.9) | 6.8 (5.5-8.4) | NS |
| 2hPG (mmol/L) | 11.1 (7.8-14.8) | 11.6 (8.2-13.0) | NS |
| HbA1c (%) | 7.6 (5.9-9.5) | 7.5 (5.9-8.9) | NS |
| HDL-c (mmol/L) | 1.3± 0.3 | 1.3± 0.3 | NS |
| LDL-c (mmol/L) | 3.0± 1.0 | 3.0± 0.7 | NS |
| ALT (u/l) | 24.3 (18.1-37.9) | 26.3 (17.5-37.8) | NS |
| TBIL (umol/l) | 13.9 (11.4-18.9) | 11.5 (9.6-15.3) | NS |
| BUN (mmol/L) | 5.4 (4.5-6.4) | 5.3 (4.4-6.6) | NS |
| TG (mmol/L) | 1.7 (1.2-2.8) | 1.6 (1.3-2.2) | NS |
| TC (mmol/L) | 5.2± 1.0 | 5.1± 0.9 | NS |
| 24hUALB (mg/24h) | 53.2 (36.5-91.9) | 72.2 (23.3-116.4) | NS |
| Cr (umol/l) | 94.7± 17.7 | 97.8± 14.2 | NS |
| FINS (μU/mL) | 14.0 (9.0-20.0) | 13.0 (9.0-18.0) | NS |
| SBP (mmHg) | 120.0 (106.3-130.0) | 120.0 (107.5-140.0) | NS |
| DBP (mmHg) | 80.0 (70.0-85.0) | 70.0 (70.0-88.5) | NS |
| HOMA-IR | 4.4 (2.7-6.9) | 4.2 (2.4-6.3) | NS |
| AIP | 2.1 (1.9-2.4) | 2.1 (1.9-2.2) | NS |
| Smoking history, n (%) | 46 (41.1%) | 12 (48%) | NS |
| Men | 45 (80.4%) | 11 (84.6%) | NS |
| Women | 1 (1.8%) | 1 (8.3%) | NS |
| Alcohol consumption, n (%) | 31 (27.7%) | 8 (32%) | NS |
| Men | 28 (50.0%) | 7 (53.8%) | NS |
| Women | 3 (5.4%) | 1 (8.3%) | NS |
| Carotid IMT(mm) | 0.71 (0.60-0.80) | 0.70 (0.62-0.80) | NS |
| Femoral IMT(mm) | 0.72 (0.60-0.80) | 0.80 (0.73-0.80) | 0.006 |
| Iliac IMT(mm) | 0.78 (0.70-0.80) | 0.80 (0.77-0.83) | 0.008 |
| FGF19* (pg/ml) | 124.8 (77.7-195.6) | 206.2 (110.2-294.3) | 0.026 |

*Log transformed before analysis. Data are means ± SD or IQR.

Abbreviation: BMI: body mass index; WHR: waist-hip ratio; FBG: fasting blood glucose; 2hPG: 2 hours postprandial blood glucose; HbA1c: hemoglobin A1c; HDL-c: high-density lipoprotein cholesterol; LDL-c: low-density lipoprotein cholesterol; ALT: alanine transaminase; TBIL: total bilirubin; BUN: blood urea nitrogen; Cr: creatinine; TC: total cholesterol; TG: triglycerides; 24hUALB: 24-hour urine microalbumin; FINS: fasting insulin; SBP: systolic blood pressure; DBP: diastolic blood pressure; HOMA-IR: Homeostasis model assessment of insulin resistance; AIP: plasma atherosclerosis index; Carotid IMT: carotid intima-media thickness; Femoral IMT: femoral intima-media thickness; Iliac IMT: iliac intima-media thickness; FGF19: fibroblast growth factor 19; SD: standard deviation; IQR: interquartile range.

**Supplementary Table 2.** Baseline clinical characteristics of subjects who completed 3-year follow-up and those who dropped out

|  | Completed follow-up (n=137) | Dropped out  (n=16) | *P* values |
| --- | --- | --- | --- |
| Age (years) | 54.0 (49.0-61.0) | 47.5 (42.0-55.0) | 0.018 |
| BMI (kg/m^2^) | 23.9(22.6-25.7) | 25.0 (23.2-26.0) | NS |
| WHR | 0.89 ± 0.05 | 0.91 ± 0.10 | NS |
| FBG (mmol/L) | 6.9 (5.8-8.0) | 8.1 (6.4-9.2) | NS |
| 2hPG (mmol/L) | 11.3 (8.1-14.4) | 12.1 (10.6-17.8) | NS |
| HbA1c (%) | 7.5 (5.9-9.2) | 8.3 (6.3-10.0) | NS |
| HDL-c (mmol/L) | 1.3± 0.3 | 1.2± 0.3 | NS |
| LDL-c (mmol/L) | 3.0± 0.9 | 2.8± 0.9 | NS |
| ALT (u/l) | 24.3 (17.9-37.7) | 26.0 (15.1-36.2) | NS |
| TBIL (umol/l) | 13.4 (10.8-18.6) | 12.6 (11.4-16.9) | NS |
| BUN (mmol/L) | 5.3 (4.4-6.4) | 5.5 (4.4-6.4) | NS |
| Cr (umol/l) | 95.3± 17.1 | 86.2± 19.1 | 0.016 |
| TG (mmol/L) | 1.6 (1.2-2.5) | 2.5 (1.5-3.9) | NS |
| TC (mmol/L) | 5.2± 1.0 | 5.5± 1.5 | NS |
| 24hUALB (mg/24h) | 54.7 (33.4-94.7) | 75.3 (13.8-116.4) | NS |
| FINS (μU/mL) | 14.0 (9.0-19.0) | 15.0 (12.0-17.0) | NS |
| SBP (mmHg) | 120.0 (107.5-130.0) | 120.0 (110.0-130.0) | NS |
| DBP (mmHg) | 78.0 (70.0-85.0) | 75.0 (70.0-80.0) | NS |
| HOMA-IR | 4.3 (2.7-6.7) | 5.4 (4.2-6.3) | NS |
| AIP | 2.1 (1.9-2.4) | 2.4 (2.0-2.6) | NS |
| Smoking history, n (%) | 58 (42.3%) | 4 (25.0%) | NS |
| Alcohol consumption, n (%) | 39 (28.5%) | 5 (31.3%) | NS |
| Carotid IMT (mm) | 0.70 (0.60-0.80) | 0.64 (0.57-0.75) | NS |
| Femoral IMT(mm) | 0.73 (0.60-0.80) | 0.77 (0.63-0.83) | NS |
| Iliac IMT (mm) | 0.80 (0.73-0.80) | 0.74 (0.64-0.78) | NS |
| FGF19* ( pg/ml) | 133.1 (80.6-219.2) | 138.1 (97.4-236.6) | NS |

*Log transformed before analysis. Data are means ± SD or IQR.

Abbreviation: BMI: body mass index; WHR: waist-hip ratio; FBG: fasting blood glucose; 2hPG: 2 hours postprandial blood glucose; HbA1c: hemoglobin A1c; HDL-c: high-density lipoprotein cholesterol; LDL-c: low-density lipoprotein cholesterol; ALT: alanine transaminase; TBIL: total bilirubin; BUN: blood urea nitrogen; Cr: creatinine; TC: total cholesterol; TG: triglycerides; 24hUALB: 24-hour urine microalbumin; FINS: fasting insulin; SBP: systolic blood pressure; DBP: diastolic blood pressure; HOMA-IR: Homeostasis model assessment of insulin resistance; AIP: plasma atherosclerosis index; Carotid IMT: carotid intima-media thickness; Femoral IMT: femoral intima-media thickness; Iliac IMT: iliac intima-media thickness; FGF19: fibroblast growth factor 19; SD: standard deviation; IQR: interquartile range.

**Supplementary Table 3.** Logistic regression analysis showing FGF19 levels at year 3 independently associated with the subAS at year 3 in men

| Variable | OR | *P* | 95% CI |
| --- | --- | --- | --- |
| 3y-FGF19 (per 1-SD increase) | 2.987 | 0.029 | 1.118-7.981 |

Abbreviation: FGF19: fibroblast growth factor 19; 3y- FGF19: serum levels of FGF19 at year 3; SD: standard deviation; subAS: subclinical atherosclerosis.

**Supplementary Figure 1.** Receiver-operating characteristic (ROC) curve for the performance of FGF19 levels at year 3 as a biomarker of subAS at year 3 in men


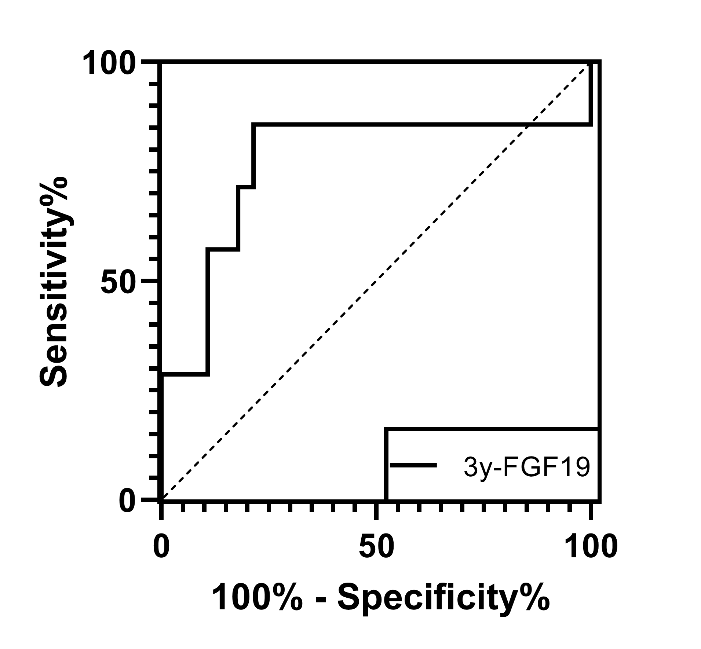

Supplement: Supplementary file 1 [file Data_Sheet_1.docx]
